# Evaluation of Forensic Luminol in Detection of Blood Stains in Instruments Following Dental Treatment

**DOI:** 10.7759/cureus.57676

**Published:** 2024-04-05

**Authors:** Akshai Senthilkumar, Vignesh Ravindran, Abirami Arthanari, Karthikeyan Ramalingam

**Affiliations:** 1 Forensic Odontology, Saveetha Dental College and Hospitals, Saveetha Institute of Medical and Technical Sciences, Saveetha University, Chennai, IND; 2 Pediatric and Preventive Dentistry, Saveetha Dental College and Hospitals, Saveetha Institute of Medical and Technical Sciences, Saveetha University, Chennai, IND; 3 Oral Pathology and Microbiology, Saveetha Dental College and Hospitals, Saveetha Institute of Medical and Technical Sciences, Saveetha University, Chennai, IND

**Keywords:** peroxidase, aminophthalhydrazide, latent stains, blood, presumptive test, chemiluminescence, blood detection, forensic luminol, luminol, forensic science

## Abstract

Background

Saliva and blood, being biological materials with a high potential for infectious transmission in dental environments, pose significant risks to dental professionals, assistants, and patients alike. Therefore, practitioners must adopt stringent security measures to ensure patient care, considering all parties as potential carriers of microorganisms capable of causing infectious diseases. Currently, various methods of disinfection and sterilization are employed to maintain the aseptic chain effectively. Having reliable methods for detecting substances in liquids, particularly body fluids, is crucial and highly convenient. Luminol, a chemiluminescent agent widely used in forensic science for detecting minute traces of blood that are invisible to the naked eye, presents itself as a valuable tool. Blood, a major bodily fluid often present in instruments following dental procedures, underscores the importance of its detection. Hence, in this study, luminol was utilized to detect blood traces in dental instruments following dental treatment, both before and after sterilization or disinfection.

Objective

Blood and saliva splashes, together with highly contagious aerosols, are always a part of dental procedures. The objective of the current study is to detect traces of blood stains on face shields, surgical instruments, and endodontic files using luminol before and after sterilization.

Materials and methods

Sample size calculation was done with G*Power software (Version 3.1.9.4, Düsseldorf, Germany), and a total of 30 instruments were selected for the study. In the present study, a total of 30 items were collected and utilized, including 14 instruments used after implant placement, 12 endodontic files employed after root canal treatment, and four face shields utilized during these procedures. Meanwhile, a freshly prepared luminol solution was applied to these instruments, and they were viewed in a dark environment both before and after sterilization procedures. Luminescence generated by luminol was observed in the instruments, indicative of the presence of blood not visible to the naked eye. Statistical analysis for both groups was done with IBM SPSS Statistics for Windows, Version 16.0 (Released 2007; SPSS Inc., Chicago, IL, USA). Intragroup comparison was done using the Friedman test, and intergroup comparison was done using the Wilcoxon signed-rank test.

Results

Blood stains and chemiluminescence were visualized in two out of 10 endodontic files (one #15 K-file and #20 K-files) and two out of four face shields. The intragroup comparison was done using the Friedman test, and it was found to be statistically significant (p < 0.05). Intergroup comparison was done using the Wilcoxon signed-rank test and was found to be statistically insignificant (p > 0.05).

Conclusion

Following sterilization and disinfection, there were no visual blood stains or chemiluminescence. Therefore, luminol was found to be effective in detecting blood stains in endodontic files, surgical instruments, and face shields, as well as in validating the sterilization and disinfection processes. Hence, sterilization in dentistry stands as a critical measure to guarantee patient safety, halt the dissemination of infections, and uphold exemplary clinical care standards.

## Introduction

In dentistry, prioritizing the safety and welfare of both dental practitioners and patients is of utmost importance [1]. A crucial part of this duty involves effectively handling and averting the spread of bodily fluid contamination throughout clinical procedures [1,2]. Clinical procedures in dental treatment often lead to the release of aerosols and the splatter of bodily fluids like blood and saliva [3]. There are several pathogens and viral particles in blood and dental aerosols that can cause infections and could be dangerous for dental professionals [4]. Following the dental treatment, the instruments used, surfaces of personal protective equipment, face masks, and protective eyewear may become contaminated by aerosols and bodily fluids [5]. The process of eliminating bacteria and spores from the surface being treated is known as sterilization. If the instruments are not properly sterilized, it could lead to cross-contamination. In dentistry, a variety of asepsis-related disinfection and sterilization techniques are used [6]. Short-term autoclaving was the most popular sterilization technique among other techniques [7]. The importance of autoclaving in dentistry is immense, as it plays a crucial role in preventing the spread of infectious diseases among patients and dental practitioners [8]. By ensuring that instruments are properly sterilized, the likelihood of cross-contamination during procedures is minimized, thereby protecting the health of patients and the safety of dental staff [9].

Contamination spills, whether they involve bodily fluids or other infectious substances, escalate the risk by creating potential avenues for harmful microorganisms to spread. These spills may occur during routine procedures or in emergencies, necessitating immediate and efficient handling to avert further contamination [10]. There exists a limited body of research examining the potential for 86-88% blood contamination in protective eyewear [11]. Chemiluminescent compounds can be used to detect bodily fluid traces, such as blood, in an instrument. Luminol is a chemiluminescent substance that is frequently employed in forensic science to detect traces of blood, even in minute quantities [12]. Due to its adaptability and convenience, this manipulation has been seamlessly integrated into the field of dentistry. Several researchers have detected and assessed blood stains using luminol on endodontic files, face shields, and within clinical operating environments [3]. Their findings concluded that even minute traces of blood were detectable by luminol [3]. The luminescence generated by luminol was effectively observed in dark environments. Luminol techniques function through the reaction between luminol and hydrogen peroxide, catalyzed by the iron (heme) in hemoglobin. This yields chemiluminescence, a phenomenon detectable through diverse methodologies.

The objective of the current study is to detect the traces of blood stains on face shields, surgical instruments, and endodontic files using luminol before and after sterilization.

## Materials and methods

Study design

This study was conducted in vitro, and approval from the Scientific Review Board was obtained from Saveetha Dental College and Hospitals, Chennai, India (approval number SRB/SDC/FORENSIC-1854/23/064). The research was carried out in the Department of Forensic Odontology and the Department of Pedodontics at Saveetha Dental College and Hospitals, Chennai, India. The study’s sample size was determined utilizing G*Power software (Version 3.1.9.4, Düsseldorf, Germany), drawing from data provided by Arruda-Vasconcelos et al. In the present study, a total of 30 instruments were carefully chosen for inclusion.

Instrument selection

**Figure 1:**
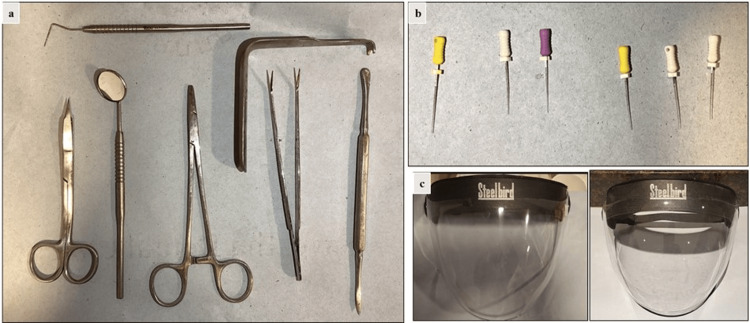
Representative images of the instruments used in this study: (a) surgical instruments used for implant placement; (b) endodontic files used for root canal treatment; and (c) face shields used for the abovementioned treatments

Surgical instruments following implant placement with upper central incisors (with adequate bone width), including mouth mirrors, periodontal probes, periosteal elevators, Austin retractors, suture needle holders, mayo surgical suture cutting scissors, and tweezers, were selected. Moreover, 2% endodontic stainless steel hand K-files of sizes 10, 15, and 20 (Mani, Inc., Utsunomiya, Japan) were selected for performing root canal therapy in the right mandibular first molar. Face shields (Steelbird visor face shields) used for the abovementioned treatments by the post-graduate trainees in their respective fields were selected and used in the present study. Figure 1 shows the representative images of the instruments used in the current study.

Sterilization procedures

Disinfection of face shields was done using isopropyl alcohol. Other instruments mentioned above were sterilized using an autoclave (121°C for 15 minutes) before the commencement of dental procedures. Following the treatment, all the instruments were washed, dried, and sterilized using an autoclave.

Treatment procedure

Implant placement was performed by a single operator from the implant surgical unit. Following the incision, the flap was reflected using a periosteal elevator and retracted using Austin’s retractor. Exploratory instruments (mouth mirror, Williams periodontal probe, and tweezer) were used to visualize and examine the implant site. After implant placement, the flap was repositioned and sutured using needle holder forceps. A total of 14 surgical instruments were selected and used in the present study.

Root canal therapy was conducted by a single postgraduate from the Department of Pediatric Dentistry. Following access cavity preparation with a round bur, patency filing was done with a #10 K-file. After working length determination with #15 K-file, initial cleaning and shaping were done using #20 K-file. A total of 12 endodontic files, which include two #10 K-files, six #15 K-files, and four #20 K-files following root canal treatment, were collected and used for the study.

The obtained instruments (n = 30) were not washed or disinfected but were utilized in the study immediately after the aforementioned treatments, with proper safety precautions maintained. The researcher wore a triple-layered mask and surgical gloves during the procedures. Following the application of luminol, the surgical and endodontic instruments were cleaned, washed, dried, and sterilized using an autoclave. Following that, disinfection of the face shields was done using isopropyl alcohol (Septodont, Saint-Maur-des-Fossés, France).

Luminol preparation

**Figure 2:**
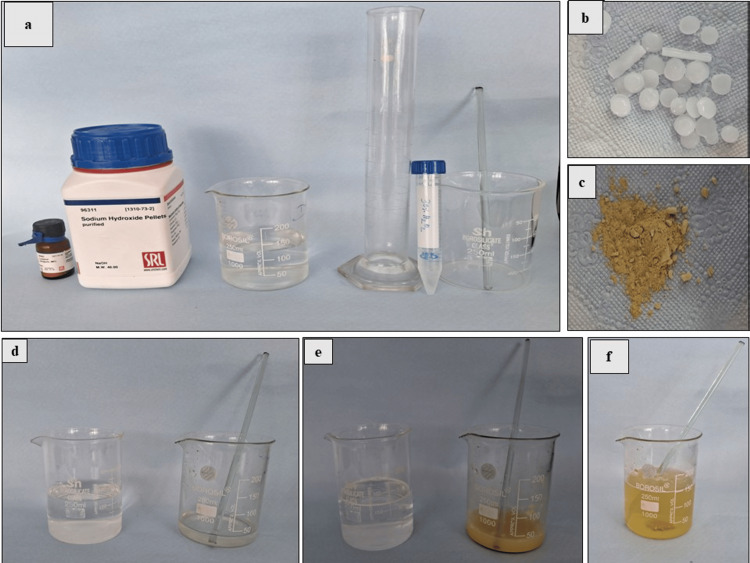
Preparation of luminol: (a) reagents and instruments used: (from left) luminol, sodium hydroxide, distilled water in a beaker, measuring cylinder, hydrogen peroxide, and beaker with stirrer; (b) 5 g of sodium hydroxide; (c) 1 g of luminol; (d) (from left) 5 g of NaOH in 500 ml of distilled water and 10 ml of 3% hydrogen peroxide solution; (e) (from left) dissolution of 5 g of NaOH in 500 ml of distilled water and 1 g of luminol in hydrogen peroxide solution; and (f) freshly prepared luminol solution (from the mixture of both solutions (e))

Luminol reagent is freshly prepared before its application at a crime scene. Commonly reported formulations in the previous literature are by Grodsky, Weber, Spruitt and coworkers, and Esperanca and coworkers. A modification of Weber’s formulation was used to prepare the luminol solution. The chemical compounds used were as follows: 3% hydrogen peroxide (Spectrum, Sisco Research Laboratories Pvt. Ltd. (SRL), Mumbai, India), 5 g of sodium hydroxide (Spectrum, Sisco Research Laboratories Pvt. Ltd. (SRL)), 1 g of luminol powder (Spectrum, Sisco Research Laboratories Pvt. Ltd. (SRL)), and 500 ml of distilled water. Moreover, 5 g of sodium hydroxide pellets were dissolved in 500 ml of distilled water in one beaker. Meanwhile, 1 g of luminol was dissolved in 10 ml of 3% hydrogen peroxide in another beaker. Then both solutions were mixed to obtain a freshly prepared solution of luminol. The solution was transferred to a spray bottle for further procedures. Figure 2 represents the preparation of the luminol solution.

Application of luminol solution

**Figure 3:**
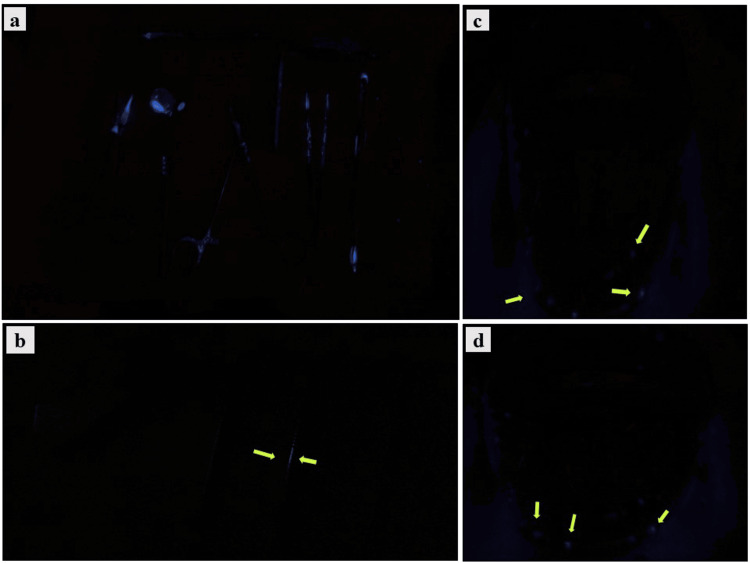
Representative images of chemiluminescence (blue color) produced by luminol of the instruments used in this study: (a) surgical instruments used for implant placement; (b) endodontic files used for root canal treatment; and (c, d) face shields used for the abovementioned treatments Arrowheads indicate the areas of chemiluminescence.

Freshly prepared luminol solution was sprayed on the selected instruments (n = 30), and they were viewed in a dark environment both before and after sterilization procedures. Luminescence produced by the luminol was noted in the instruments, which was due to the presence of non-visible blood, and photographs were taken using a digital single-lens reflex (DSLR) camera without flash. Figure 3 represents the chemiluminescence observed in the instruments.

Scoring criteria

The selected instruments were given scores according to the scoring criteria before and after the sterilization procedures (Table 1). Score 1 indicates the absence of a bloodstain that is visible to the naked eye or under luminol, and Score 2 indicates the presence of a bloodstain that is visible to the naked eye or under luminol. The obtained scores were tabulated using Microsoft Excel (Microsoft Corporation, Redmond, WA, USA), and statistical analysis was done using IBM SPSS Statistics for Windows, Version 16.0 (Released 2007; SPSS Inc., Chicago, IL, USA).

**Table 1:** Descriptive statistics of the instruments used in the study

Before sterilization					After sterilization			
Instruments used	Before luminol		After luminol		Before luminol		After luminol	
	Instruments with visible blood stains	Instruments without blood	Instruments with chemiluminescence	Instruments without chemiluminescence	Instruments with visible blood stains	Instruments without blood	Instruments with chemiluminescence	Instruments without chemiluminescence
Surgical instruments (n = 14)	14	0	14	0	0	0	0	0
Endodontic instruments (n = 12)	2	10	2	10	0	0	0	0
Face shields (n = 4)	0	4	2	2	0	0	0	0

Statistical analysis

The intragroup comparison was done using the Friedman test at a significance level of 5%. The Wilcoxon signed-rank test was done for comparison between the groups before and after sterilization using IBM SPSS Statistics for Windows, Version 16.0.

## Results

All the selected instruments following dental treatments were subjected to luminol application without any sterilization or disinfection. After luminol application, all the surgical instruments contained blood stains and also exhibited chemiluminescence, which was visible to the naked eye. Blood stains and chemiluminescence were visualized in two out of 10 endodontic files (one #15 K-file, and #20 K-files) and two out of four face shields. Following sterilization and disinfection, there were no visual blood stains or chemiluminescence. Table 1 indicates the descriptive statistics of the instruments used in the present study.

The intragroup comparison was done using the Friedman test. The Friedman test was found to be statistically significant as the p-value was less than 5%. Intergroup comparison was done using the Wilcoxon signed-rank test. The Wilcoxon signed-rank test was not statistically significant. The non-sterilized group (ns) had a score of 0.083, and the sterilized group had a score of 1 (p > 0.05). Table 2 displays the results of the intergroup comparison subsequent to statistical analysis.

**Table 2:** Statistical results of the intergroup comparison table (Wilcoxon signed-rank test)

Intergroup comparison (Wilcoxon signed-rank test)	p-value
Pre-sterilization group	0.083
Post-sterilization group	1

## Discussion

After the application of luminol, all the surgical instruments contained blood stains and also exhibited chemiluminescence, which was visible to the naked eye, whereas blood stains and chemiluminescence were visualized in 20% of the endodontic files (one #15 K-file, and #20 K-files) and in 50% of the face shields. Following sterilization and disinfection, there were no visual blood stains or chemiluminescence.

Luminol is 5-Amino-2, 3-dihydro-1, 4-phthalazinedione, or simply called 3-aminophthalhydrazide, used for decades for detecting hidden bloodstains [12]. The high sensitivity, ease of preparation, and affordability of luminol have made it a popular method in the field of forensics. A freshly prepared luminol solution can be sprayed directly on the suspected areas for the detection of blood. Luminol solutions can be prepared either by Grodsky or Weber formulations [12,13]. A modulation in Weber’s formulation led to the rise of two methods of preparation by Spruitt and coworkers and Esperanca and coworkers [12]. In the present study, Weber’s formulation was used for the preparation of luminol reagent. This study used forensic luminol to detect blood contamination in the surgical instruments, endodontic files, and face shields following minor oral surgical and endodontic procedures, before and after the sterilization process.

Following luminol application to an object or surface, there could be an emission of a bright blue color, even in the presence of minute traces of blood (chemiluminescent property), and this can be recorded with the help of high-resolution cameras. In our study, the chemiluminescence was captured by a DSLR camera (Canon EOS R50, 28 mm, Canon Inc., Ota City, Tokyo, Japan). The camera settings were modified to capture images in a dark environment without a flash (f/5, 1 second, ISO 6400, Exp: 0, no flash).

Luminol, phenolphthalein, and leucomalachite green staining are the most preferred methods in the literature for detecting blood stains that are not readily visible to the naked eye [14]. Among them, the luminol test is regarded as one of the most well-known tests in the field of forensics. Luminol exhibits chemiluminescence properties in the presence of hemoglobin [15]. A dark environment is required to visualize the chemiluminescence produced by luminol [16]. In this study, the chemiluminescent property was visualized in a dark operating room in the Department of Pediatric Dentistry. The chemiluminescence is interfered with by many factors, such as temperature, pH, nature of the surface, chemicals, food and drinks, and the concentration of reagent used [17]. Previous literature suggests that sodium hypochlorite, which is used as an irrigant during instrumentation of the root canals in an endodontic treatment, has the potency to alter the efficacy of luminol [13]. During an endodontic treatment of an infected pulp or periapical tissues, there are higher chances of vascular bleaching. Blood from these areas could contact the endodontic files and might become a source of infection if not properly sterilized. Arruda-Vasconcelos et al. evaluated endodontic files immediately after endodontic treatment and concluded that luminol was effective in detecting the blood stains that were unable to be detected on visual examination [18]. It can be attributed to the fact that luminol can detect the presence of blood even at minute traces. This statement is in accordance with our study. The selected endodontic (2%) stainless steel k-files of different diameters used in our study were evaluated for the presence of blood stains following the cleaning and shaping procedures. Blood stains in these endodontic files were only visible after the application of luminol. This can also be attributed to the fact that the utilization of sodium hypochlorite during instrumentation does not alter the efficacy of luminol in the detection of blood.

Luminol has recently been used in a study to assess the efficacy of disinfection in a medical setting. Englehardt et al. reported that luminol could find imperceptibly faint blood traces on every specimen board positioned around the surgical area to collect splattered and aerosolized blood [19].

During minor oral surgical procedures, there is a possibility that the high frequency of aerosolized and splattered blood droplets could increase the risk of blood-borne infection transmission [11]. Ishihama et al. concluded that in 90% of cases, aerosolized and splattered blood contamination was the result of oral surgical procedures utilizing rotary instruments. Additionally, they noticed that over 50% of blood contamination is invisible to the naked eye and could only be discovered through indirect blood detection methods. The surface of the clinical operative field, surgical kits, and protective eyewear could also be contaminated with bodily fluids and aerosols [20]. A study by Yamada et al. stated that surgeons performing high-speed dental procedures ran the risk of acquiring blood-borne infections [21].

In our study, surgical instruments obtained from the implant surgical unit contained blood stains that were evident to the naked eye, and also, following the application of luminol, chemiluminescence was observed in all fourteen surgical instruments. Face shields used during implant placement and root canal treatment were collected and used in this study. Aerosol splashes were visible to the naked eye, whereas the presence of blood was detected in two of the face shields only after the application of luminol. This finding was congruent with a study by Al-Eid et al., where it was revealed that blood contamination was found in every item used by clinical staff, as well as in eyewear and chest drapes, except head caps and shoe covers [22].

Sterilization is a procedure in which all the pathogens, including their spore forms, are destroyed. Over the years, physical means of sterilization have been the most preferred method in the field of dentistry [23]. Among them, autoclaving the used dental instruments is the preferred mode of physical sterilization (121°C for 15 minutes). Hence, in this study, all the surgical and endodontic instruments were disinfected, washed, dried, and then autoclaved.

Chemical disinfection using isopropyl alcohol is found to be effective against viral pathogens [24]. The face shields used in the current study were disinfected using isopropyl alcohol. Septodont dimenol spray was used as a disinfectant to clean the face shields. The results of the present study revealed that the p-value (>0.05) was not statistically significant. This could be due to the limited sample size. However, luminol was found to be effective in detecting blood stains, even at minute traces. Following the sterilization and disinfection procedures, all the instruments were free from the presence of bodily fluids. This can be attributed to the fact that thorough sterilization and disinfection procedures are required for dental operating instruments and the dental operating field to prevent cross-contamination of infections and bodily fluids.

The longevity of the luminol solution was not validated. Differences in the efficacy of various sterilization methods were not evaluated. Instruments were visualized immediately after treatment; hence, any delay in the effectiveness of luminol following different time intervals of treatment should be evaluated. Future research should consider the limitations and focus on more parameters and various forensic environments that can be used to assess the luminol solution.

## Conclusions

Within the limitations of the present study, it can be concluded that luminol was effective in detecting the presence of blood, even at minute traces. The use of luminol was also validated for the sterilization and disinfection procedures. Following sterilization and disinfection, there were no visual blood stains or chemiluminescence. Hence, sterilization in dentistry stands as a critical measure to guarantee patient safety, halt the dissemination of infections, and uphold exemplary clinical care standards.
